# Impact of Nasopharyngeal FilmArray Respiratory Panel Results on Antimicrobial Decisions in Hospitalized Patients

**DOI:** 10.1155/2018/9821426

**Published:** 2018-06-13

**Authors:** Kenneth K. Sakata, Natalya Azadeh, Anjuli Brighton, M'hamed Temkit, Christine L. Klassen, Thomas E. Grys, Holenarasipur R. Vikram

**Affiliations:** ^1^Division of Pulmonary Medicine, Mayo Clinic, Phoenix, AZ, USA; ^2^Division of Pulmonary and Critical Care Medicine, Mayo Clinic, Rochester, MN, USA; ^3^Division of Health Sciences and Research, Mayo Clinic Arizona, Scottsdale, AZ, USA; ^4^Division of General Internal Medicine, Mayo Clinic, Rochester, MN, USA; ^5^Department of Laboratory Medicine and Pathology, Mayo Clinic, Phoenix, AZ, USA; ^6^Division of Infectious Diseases, Mayo Clinic, Phoenix, AZ, USA

## Abstract

**Objective:**

To determine whether results of the nasopharyngeal FilmArray respiratory panel (NP-FARP) influenced antibiotic decisions.

**Methods:**

We reviewed the medical records of nonintensive care unit (ICU) inpatients that had an NP-FARP performed at our institution between June 2013 and June 2014. The inpatient records were reviewed 48 hours after the NP-FARP for the following data: demographic information; NP-FARP, serum procalcitonin, and methicillin-resistant *Staphylococcus aureus* nasal swab (MRSA NS) results; antibiotics prior and post-48 hours of the NP-FARP result; and the current immunosuppression status. Clinical outcome data were not obtained. Patients were categorized into those who had a positive (+) or a negative (−) NP-FARP. We further subdivided these two categories into groups A, B, and C based on the antibiotic modifications 48 hours after their NP-FARP result. Group A included patients who were never initiated on antimicrobial therapy. Patients whose antibiotics were discontinued or deescalated were placed in group B. Patients with antibiotic escalation or continuation without change constituted group C. We compared and analyzed groups A, B, and C in the (+) and (−) NP-FARP cohorts.

**Results:**

A total of 545 patients were included. There were 143 (26%) patients with positive and 402 (74%) patients with negative NP-FARPs. Comparison of groups A, B, and C between those with a (+) and (−) NP-FARP were as follows: (+) A and (−) A, 28/143 (20%) and 84/402 (21%); (+) B and (−) B, 59/143 (41%) and 147/402 (37%); and (+) C and (−) C, 56/143 (39%) and 171/402 (43%), respectively. We found no statistically significant differences between groups (+) A versus (−) A, (+) B versus (−) B, and (+) C versus (−) C with respect to age, gender, MRSA NS result, procalcitonin result, or concurrent immunosuppression.

**Conclusion:**

In non-ICU inpatients, NP-FARP alone or in combination with procalcitonin or MRSA NS did not influence antibiotic decisions during the study period.

## 1. Introduction

Respiratory tract infections remain the most common illness in humans for which urgent medical care is sought [1]. Early differentiation between bacterial infection and a self-limited viral infection is essential and continues to pose a major diagnostic challenge. Presenting symptoms of a viral respiratory illness may be indistinguishable from bacterial infections. Approximately three-quarters of all antibiotic doses are prescribed for acute viral respiratory tract infections [2]. Unnecessary antimicrobial therapy leads to increased healthcare costs, adverse events (such as *Clostridium difficile* infections), and the emergence of multidrug-resistant organisms [3]. A recent study describing the incidence of the antibiotic-associated adverse drug effect for adult inpatients receiving systemic antibiotic therapy showed that up to 20% of patients developed at least 1 antibiotic-associated adverse drug effect [4]. Twenty percent of nonclinically indicated antibiotic regimens were associated with an adverse drug effect, including 7 cases of *C. difficile* infection [4].

The nasopharyngeal FilmArray respiratory panel (NP-FARP) (BioFire Diagnostics, Salt Lake City, UT) was the first multiplex molecular panel approved in 2011 by the United States Food and Drug Administration for the detection of both bacterial and viral respiratory pathogens in nasopharyngeal swabs and was brought into our institution in February 2013. The NP-FARP targets 17 viruses and subtypes and 3 bacteria with high sensitivity and specificity. It has a fully automated sample-to-answer workflow with a turnaround-time of approximately 1 hour [5]. The performance characteristics, simplified workflow, and rapid turnaround-time have allowed its implementation in a wide range of laboratories and clinical settings.

We hypothesized that a positive NP-FARP for any of the 17 viral pathogens in the context of an absence of data supporting a bacterial infection would persuade clinicians to not initiate, discontinue, or deescalate antibiotic therapy. The primary aim of this retrospective observational study was to determine if the NP-FARP influenced antibiotic decisions. We also looked into whether the adjunctive use of serum procalcitonin, methicillin-resistant *Staphylococcus aureus* nasal swab (MRSA NS), or an immunosuppressed host impacted antibiotic decisions.

## 2. Materials and Methods

We reviewed the electronic medical records of all non-ICU inpatients that had an NP-FARP performed between June 1, 2013 and June 30, 2014. Inpatient progress notes and medication records were reviewed 48 hours after NP-FARP was performed to determine whether NP-FARP results led to a change in antibiotic regimens. When performed within 48 hours of the NP-FARP, procalcitonin was recorded as either not done or negative, and the MRSA NS was recorded as either not done, positive, or negative. For patients who were discharged < 48 hours after their NP-FARP, their discharge summary medication list was used. Outpatients, ICU patients, and patients with a positive procalcitonin (>0.25 *µ*g/L) were excluded. Those who tested positive for any of the 3 atypical bacterial respiratory pathogens by NP-FARP (*Legionella* sp., *Chlamydia* sp., and *Mycoplasma* sp.) were excluded. Prophylactic antibiotic regimens in immunosuppressed patients were not considered in our analysis. Clinical outcomes were not assessed.

### 2.1. Definitions

#### 2.1.1. Positive and Negative NP-FARP

A positive (+) NP-FARP identified a viral pathogen by multiplex PCR, and a negative (−) NP-FARP did not.

#### 2.1.2. Antibiotic Decisions

All antibiotic decisions were documented based on what transpired within 48 hours of the NP-FARP result. Discontinuation of antibiotics was defined as the cessation of all empiric antimicrobial therapy. Narrowing of the antimicrobial spectrum (i.e., changing from a third-generation cephalosporin to a first-generation cephalosporin) or any decrease in the number of empiric antibiotics was termed deescalation. Patients who had their antimicrobial spectrum broadened (i.e., changing from a first-generation cephalosporin to a third-generation cephalosporin) or had any increase in the number of antibiotics after their NP-FARP results were referred to as escalation. Those who remained on the same antimicrobial regimen after the NP-FARP results were considered to continue therapy. Patients who were never initiated on antimicrobial therapy were labeled as no antibiotics and placed into group A. Discontinuation and deescalation were placed in group B. Escalation and continuation of therapy constituted group C.

#### 2.1.3. Immunosuppression

Immunosuppression was defined as any hematologic malignancy, solid-organ or bone marrow transplantation, and/or receipt of any of the following immunosuppressive therapies at the time of the NP-FARP: cancer chemotherapy, systemic corticosteroids, therapeutic interferon preparations, tumor necrosis factor and interleukin inhibitors, azathioprine, methotrexate, calcineurin inhibitors, or mycophenolate mofetil.

#### 2.1.4. Empiric Antimicrobial Therapy

Empiric antimicrobial therapy was defined as any antibiotic(s) apart from its prophylactic antibiotic regimen.

#### 2.2. Statistical Methods

Our study patients were first divided into cohorts with a positive or negative NP-FARP. We further subdivided these cohorts into groups A, B, and C based on the patient's antibiotic modification 48 hours after their NP-FARP result. We compared and analyzed groups A, B, and C in the (+) and (−) NP-FARP cohorts.

The descriptives consist of frequencies and proportions for categorical variables, median, first and third quartiles, minimum, maximum, and range for continuous variables. The group comparisons were conducted using the Wilcoxon rank sum test to test for population mean shift between two groups for continuous variables. The Pearson chi-square and the Fisher's exact test were used in the presence of low frequencies to test for univariate associations between the categorical values. The significance level was at 0.05. Statistical analyses were performed using the statistical software packages SAS version 9.3 (SAS Institute, Cary, NC) and R version 3.1.2.

The study was approved by the Mayo Clinic Institutional Review Board.

## 3. Results

**Figure 1:**
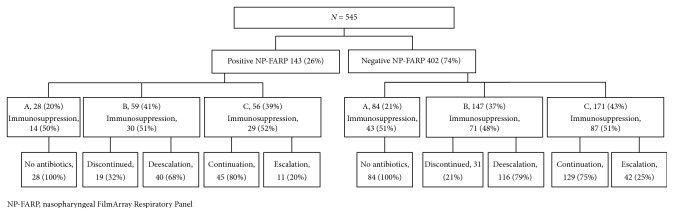
Flow diagram depicting positive and negative NP-FARP cohorts with subsequent antibiotic decisions. NP-FARP, nasopharyngeal FilmArray respiratory panel.

A total of 545 patients were included in the study analysis. There were 143 (26%) and 402 patients (74%) with positive and negative NP-FARPs, respectively. Comparison of groups A, B, and C between those with (+) and (−) NP-FARPs is detailed in Figure 1. In the total cohort, 64 (18%) and 226 (41%) patients underwent procalcitonin and MRSA NS testing, respectively. Within the group that had a positive NP-FARP, 17 (12%) and 53 (37%) patients had procalcitonin and MRSA NS performed, respectively. In the negative NP-FARP group, 47 (12%) and 173 (43%) patients had procalcitonin and MRSA NS performed, respectively.

Basic demographic information, MRSA NS, and procalcitonin results, and underlying immunosuppression in groups A, B, and C are described in Table 1. We found no statistically significant differences between groups (+) A versus(−) A, (+) B versus (−) B, and (+) C versus (−) C with respect to age, gender, MRSA NS result, procalcitonin result, or concurrent immunosuppression.

Among groups B and C, the most common antibiotic regimens started prior to NP-FARP and then discontinued (group B) or added (group C) 48 hours after NP-FARP are listed in Table 2. In group B, the most common antibiotic regimen started prior to NP-FARP was the combination of anti-MRSA + *β*-lactam + atypical pathogen coverage. The most common antibiotic regimen discontinued after NP-FARP was the combination anti-MRSA + *β*-lactam. The most common antibiotic regimen started prior to NP-FARP in group C was atypical pathogen coverage, which was also the most common antibiotic added after the NP-FARP result.

Among group B, there were a total of 104 patients in whom antibiotics with anti-MRSA activity were discontinued. Of these, 67 (64%) patients had a concurrent negative MRSA NS, 6 (6%) patients had a concurrent positive MRSA NS, and 31 (30%) patients did not have an MRSA NS performed. Conversely, there were 12 patients in whom anti-MRSA antibiotic therapy was added: 8 (67%) patients did not have an MRSA NS performed, 3 (25%) patients had a negative MRSA NS, and 1 (8%) patient had a positive MRSA NS.

From group C, 150 of 227 (66%) patients had documented clinical reasons for their antibiotic continuation or escalation. The most frequently cited indications were clinical/radiographic deterioration or persistent signs and symptoms of infection. Seventy-one (31%) patients documented an identified pathogen by sputum culture or bronchoalveolar lavage, and 25 (11%) patients had an additional concurrent nonrespiratory tract infection.

## 4. Discussion

We hypothesized that, in non-ICU inpatients with a respiratory tract infection and the absence of evidence suggesting a bacterial etiology (i.e., positive procalcitonin or a positive bacterial culture), the identification of a viral pathogen by NP-FARP should prompt clinicians to consider withholding initiation, discontinuing, or deescalating antibiotics. ICU patients and those with a positive procalcitonin were excluded from our analysis because we felt that withholding, discontinuation, or deescalation of antibiotics would be unlikely regardless of the NP-FARP results. The similar proportions of patients between groups (+) A and (−) A, (+) B and (−) B, and (+) C and (−) C suggest that antibiotic decisions were made irrespective of NP-FARP results. Between each group, age and gender differences did not meet the statistical significance. Additional variables including MRSA NS, procalcitonin, or underlying immune suppression did not reach statistical significance, suggesting that these factors did not influence antibiotic decisions either. Therefore, NP-FARP used alone or in combination with adjunctive MRSA NS or procalcitonin does not appear to influence antibiotic decisions during the study time frame. Subgroup analysis revealed that the majority of patients who had their anti-MRSA antibiotic discontinued had a concurrent negative MRSA NS. A prior study from our institution showed that MRSA NS had a negative predictive value of 99% for excluding MRSA pneumonia [6]. The local awareness of the results of this study likely influenced a significant number of antimicrobial discontinuation decisions in group B when MRSA NS was negative.

Several studies have analyzed the clinical and economic impacts of multiplex respiratory testing [7]. In one study, comparison of outcomes of adult patients who tested positive for respiratory viruses across 2 influenza seasons was undertaken [8]. During the first season, conventional methods (viral culture, rapid antigen testing, and direct fluorescent antibody testing) were used and were compared to FARP obtained from the nasopharynx or bronchoalveolar lavage during the second season. FARP led to a significant decrease in the time to diagnosis of influenza (1.7 versus 7.7 hours) and noninfluenza viruses (1.5 versus 13.5 hours) compared to conventional methods. The study also found significantly lower odds for admission, duration of antimicrobial use, length of stay, and number of chest radiographs when influenza virus was detected by FARP [8]. Two additional studies in pediatric patients demonstrated better sensitivity with NP-FARP, lower likelihood of antimicrobial therapy beyond 48 hours, decreased length of hospital stay, and isolation time [9, 10]. A single-center randomized controlled trial in adult patients presenting with acute respiratory illness found that point-of-care molecular testing with nasopharyngeal and oropharyngeal FARP led to shorter courses of antibiotics (<48 hours), shorter length of stay, and improved antiviral use for patients with influenza when compared to those in the control group who were tested by PCR assays at the discretion of the clinician [11]. Overall, there was no statistical difference in the mean duration of antibiotics and the proportion of patients who received antibiotics.

Another prospective randomized trial assessed the feasibility of using procalcitonin algorithms with nasopharyngeal and oropharyngeal FARP testing to guide antibiotic administration [12]. The investigators found no significant differences in overall antibiotic exposure or adverse events between the intervention and standard-of-care groups. Although the duration of antibiotic therapy was similar in the 2 groups, subgroup analyses of patients with the lowest risk for bacterial infection (patients with a positive FARP and a low procalcitonin level) revealed that significantly fewer patients were discharged on antibiotics and received a shorter duration of therapy compared to the nonintervention group. Importantly, investigators used a proactive communication strategy of text paging results while also simultaneously emailing the procalcitonin algorithm to providers. Such a strategy may not be feasible on a long-term basis outside the scope of a clinical trial, but some version of proactive communication will be critical to translate lab results to stewardship success.

Cost is an important consideration when utilizing novel testing strategies on a large scale. Multiplex PCR testing was the least expensive approach when compared to shell vial culture and direct fluorescent antibody testing when the prevalence of respiratory viral illness was >11% [13]. Multiplex PCR testing in the emergency room for children with influenza was cost-effective when compared with direct fluorescent antibody and traditional PCR [14]. The lab cost of each NP-FARP cartridge is approximately $125.00 USD at our institution.

Multiplex panels such as NP-FARP have several advantages and limitations. Advantages include excellent sensitivity, rapid result availability, detection of a multitude of respiratory viruses from a single sample, and ease of testing in the laboratory. They will allow better epidemiological descriptions of pathogens in a given hospital or community. They offer potential benefits such as antibiotic deescalation, shorter hospital stay, and decrease in the use of other invasive procedures for sample acquisition. Multiplex testing can identify other treatable atypical bacterial pathogens (*Chlamydia*, *Mycoplasma*, and *Bordetella pertussis*) which might otherwise be missed. Immunocompromised patients may shed respiratory viruses for a prolonged duration, and multiplex testing can identify such patients who will need to be placed in droplet isolation. However, the test result is only as good as the specimen submitted; a sample from the nose (instead of the nasopharynx) will provide a false-negative result. Viral detection will not distinguish between recent and active infection and may provide a false sense of security while masking an underlying nonviral infection as the cause of the patient's illness. Most panels do not allow for customizable ordering and testing for specific viruses and can be expensive.

There are several limitations of our study. This was a single-center retrospective observational study. Antibiotic decisions were entirely made by the clinical team, were not protocol driven, and were based on passive reporting of results to the chart since no proactive communication occurred. Patient outcomes were not recorded, and thus, we cannot comment on whether continuation, escalation, or discontinuation of antimicrobials resulted in either beneficial or adverse consequences. Impact on hospital length of stay was not determined. We did not differentiate the upper and lower respiratory tract infections in our cohort. This pilot study provided us with preliminary information pertaining to current practices and attitudes based on which future studies involving specific algorithm-driven interventions and patient outcomes can be designed. NP-FARP and procalcitonin became available at our institution in February and August 2013, respectively, and hence, providers may not have had adequate exposure and experience to fully understand and trust the utility of these tests in routine clinical practice during the study period (June 2013 to June 2014). Only 18% and 41% of patients in the total cohort, 12% and 37% of patients in the (+) NP-FARP, and 12% and 43% of patients in the (−) NP-FARP groups underwent PCT and/or MRSA NS testing, respectively. Hence, the exact contribution of each of these individual tests towards antimicrobial decisions cannot be ascertained. On the contrary, a study from our institution demonstrating an excellent negative predictive value of MRSA NS appears to have influenced provider's decision to discontinue anti-MRSA antibiotics if MRSA NS was negative with its widespread institutional publicity [6]. Repeating the current study at the present time utilizing a testing algorithm may provide us with different results.

How can the results of this study and others be utilized to improve patient outcomes and decrease unnecessary prescription of antibiotics in this era of rapidly escalating antimicrobial resistance? First, healthcare providers have to be educated about the indications for and appropriate utilization of noninvasive tests such as procalcitonin, NP-FARP, and MRSA NS to determine if a given patient has a self-limiting viral infection or another process that requires antimicrobial therapy. Algorithms involving these tests should be developed and validated in various populations (such as children, the elderly, and the immune suppressed) so that clinicians can feel confident about discontinuing or deescalating antibiotics in the absence of a bacterial infection. Such protocols will also prevent indiscriminate testing with multiplex PCR, as the cost per test is still expensive. Second, institutions should invest in a robust and all-encompassing antimicrobial stewardship program that carefully monitors antimicrobial usage, restricts indiscriminate use of broad spectrum antibiotics, and provides real-time feedback to providers. Stewardship programs can also provide guidance to clinicians regarding proper utilization of NP-FARP, procalcitonin, and other noninvasive tests and enforce early deescalation and discontinuation of antimicrobials in the appropriate clinical setting.

## 5. Conclusion

In our noninterventional retrospective observational study, NP-FARP alone, or in combination with procalcitonin, did not appear to influence antibiotic decisions. A negative MRSA NS appeared to have a stronger influence than positive NP-FARP as it pertains to deescalation and/or discontinuation of antibiotics. Incorporation of validated algorithms for appropriate test utilization, provider education, and antimicrobial stewardship program involvement to monitor and intervene when necessary can mitigate unnecessary NP-FARP testing and unwarranted antimicrobial therapy.

## Figures and Tables

**Table 1:** Demographics, MRSA NS, procalcitonin, and immunosuppression in groups A, B, and C.

	Group A				Group B				Group C			
	+A	−A	Total	*p* value	+B	−B	Total	*p* value	+C	−C	Total	*p* value
*N*	28	84	112		59	147	206		56	171	227	

Age				0.11^1^				0.36^1^				0.61^1^
Median	60.0	68.0	66.0		71.0	68.0	69.0		68.0	69.0	68.0	

Gender				0.44^2^				0.94^2^				0.18^2^
Female	17 (60.7%)	44 (52.4%)	61 (54.5%)		24 (40.7%)	59 (40.1%)	83 (40.3%)		33 (58.9%)	83 (48.5%)	116 (51.1%)	
Male	11 (39.3%)	40 (47.6%)	51 (45.5%)		35 (59.3%)	88 (59.9%)	123 (59.7%)		23 (41.1%)	88 (51.5%)	111 (48.9%)	

MRSA NS				0.43^3^				0.34^3^				0.67^3^
Not done	26 (92.9%)	67 (79.8%)	93 (83.0%)		31 (52.5%)	69 (46.9%)	100 (48.5%)		35 (62.5%)	94 (55.0%)	129 (56.8%)	
Negative	2 (7.1%)	16 (19.0%)	18 (16.1%)		26 (44.1%)	76 (51.7%)	102 (49.5%)		19 (33.9%)	68 (39.8%)	87 (38.3%)	
Positive	0 (0.0%)	1 (1.2%)	1 (0.9%)		2 (3.4%)	2 (1.4%)	4 (1.9%)		2 (3.6%)	9 (5.3%)	11 (4.8%)	

Procalcitonin				0.37^2^				0.52^2^				0.89^2^
Not done	25 (89.3%)	69 (82.1%)	94 (83.9%)		48 (81.4%)	125 (85.0%)	173 (84.0%)		53 (94.6%)	161 (94.2%)	214 (94.3%)	
Negative	3 (10.7%)	15 (17.9%)	18 (16.1%)		11 (18.6%)	22 (15.0%)	33 (16.0%)		3 (5.4%)	10 (5.8%)	13 (5.7%)	

Immunosuppression				0.91^2^				0.81^2^				0.91^2^
No	14 (50.0%)	41 (48.8%)	55 (49.1%)		29 (49.2%)	75 (51.0%)	104 (50.5%)		27 (48.2%)	84 (49.1%)	111 (48.9%)	
Yes	14 (50.0%)	43 (51.2%)	57 (50.9%)		30 (50.8%)	72 (49.0%)	102 (49.5%)		29 (51.8%)	87 (50.9%)	116 (51.1%)	

MRSA NS, methicillin-resistant *Staphylococcus aureus* nasal swab; ^1^Wilcoxon; ^2^chi-square; ^3^Fisher's exact.

**Table 2:** Common antimicrobial regimens utilized to initiate (groups B and C), discontinue (group B), or escalate (group C) therapy in patients with positive (+) or negative (−) NP-FARP.

Group (+) B	
Antibiotics started	59
Anti-MRSA + *β*-lactam + atypical pathogen coverage	19
Atypical respiratory pathogen	14
*β*-lactam + atypical pathogen coverage	10
Antibiotics discontinued	59
Atypical respiratory pathogen	16
Anti-MRSA + *β*-lactam	14
*β*-lactam	9
Anti-MRSA	6

Group (−) B	
Antibiotics started	147
Anti-MRSA + *β*-lactam + atypical pathogen coverage	54
*β*-lactam + atypical pathogen coverage	29
*β*-lactam	27
Antibiotics discontinued	147
*β*-lactam	43
Anti-MRSA + *β*-lactam	43
Anti-MRSA	20

Group (+) C	
Antibiotics started	56
Atypical pathogen coverage	18
*β*-lactam	11
Anti-MRSA + *β*-lactam + atypical pathogen coverage	8
Antibiotics escalated	11
Anti-MRSA + *β*-lactam	2
*β*-lactam	2
Atypical pathogen coverage	2

Group (−) C	
Antibiotics started	171
Atypical pathogen coverage	49
*β*-lactam	27
*β*-lactam + atypical pathogen coverage	23
Anti-MRSA + *β*-lactam + atypical pathogen coverage	20
Anti-MRSA + *β*-lactam	18
Antibiotics escalated	42
Atypical pathogen coverage	12
*β*-lactam	11
Anti-MRSA	5

MRSA, methicillin-resistant *Staphylococcus aureus*.

## Abbreviations

HCAP: Healthcare-associated pneumonia
ICU: Intensive care unit
MRSA NS: Methicillin-resistant *Staphylococcus aureus* nasal swab
NP-FARP: Nasopharyngeal FilmArray respiratory panel.
